# Mutant *BCL11B* in a Patient With a Neurodevelopmental Disorder and T-Cell Abnormalities

**DOI:** 10.3389/fped.2020.544894

**Published:** 2020-10-19

**Authors:** Sai Yang, Qingyun Kang, Yanqi Hou, Lili Wang, Liping Li, Shulei Liu, Hongmei Liao, Zhenhua Cao, Liming Yang, Zhenghui Xiao

**Affiliations:** ^1^Department of Neurology, Hunan Children's Hospital, Changsha, China; ^2^Running Gene Inc., Beijing, China; ^3^Research Institute of Pediatrics, Hunan Children's Hospital, Changsha, China; ^4^Hunan Provincial Key Laboratory of Children's Emergency Medicine, Hunan Children's Hospital, Changsha, China

**Keywords:** BCL11B, neurodevelopmental disease, immunodeficiency, developmental disorder, immune system abnormalities

## Abstract

**Background:** *BCL11B* encodes B-cell lymphoma/leukemia 11B, a transcription factor that participates in the differentiation and migration of neurons and lymphocyte cells. *De novo* mutations of *BCL11B* have been associated with neurodevelopmental disorder and immunodeficiency, such as immunodeficiency 49 (IMD49) and intellectual developmental disorder with speech delay, dysmorphic facies, and T-cell abnormalities (IDDSFTA). However, the pathogenesis of the neurodevelopmental disorder and T-cell deficiency is still mysterious. The strategy to distinguish these two diseases in detail is also unclear.

**Methods:** A patient with unique clinical features was identified. Multiple examinations were applied for evaluation. Whole-exome sequencing (WES) and Sanger sequencing were also performed for the identification of the disease-causing mutation.

**Results:** We reported a 17-month-old girl with intellectual disability, speech impairment, and delay in motor development. She presented with mild dysmorphic facial features and weak functional movement. MRI indicated the abnormal myelination of the white matter. Immunological analysis showed normal levels of RTEs and γδT cells but a deficiency of naive T cells. Genetic sequencing identified a *de novo* heterozygous frameshift mutation c.1192_1196delAGCCC in *BCL11B*.

**Conclusions:** An IDDSFTA patient of East Asian origin was reported. The unreported neurological display, immunophenotype, and a novel disease-causing mutation of the patient extended the spectrum of clinical features and genotypes of IDDSFTA.

## Introduction

*BCL11B* gene encodes the transcription factor B-cell leukemia, which regulates the differentiation, proliferation, and apoptosis of T lymphocytes. The encoded protein, BCL11B, is a zinc finger transcription factor that modulates T-cell receptors through regulating the DNA-binding transcription to direct the migration of hematopoietic progenitors (1). It also monitors the development of group 2 innate lymphoid cells. (2). The absence of functional BCL11B protein in mice blocks the T-cell lineage at the CD4^−^CD8^−^ double-negative stage, affecting the differentiation and function of thymic lymphocytes (3). BCL11B also participates in the development of multiple systems, including neurogenesis of various neuronal subtypes (4), regeneration of epithelial cells (5), and formation of ameloblasts (6).

Heterozygous mutations of *BCL11B* have been identified in some patients with developmental disorders and immunodeficiency. Immunodeficiency 49 (IMD49) is the first identified congenital disease caused by *BCL11B* mutations, characterized by severe immunodeficiency with dysmorphic features, skin abnormalities, and global developmental delay. Intellectual developmental disorder with speech delay, dysmorphic facial features, and T-cell abnormalities (IDDSFTA) is a newly identified type of BCL11B-associated congenital malformation, which overlaps with IMD49 in some features including feeding difficulties and autistic features (7). However, symptoms of IDDSFTA are relatively moderate.

Here, we report a patient with IDDSFTA caused by a *de novo* heterozygous mutation in *BCL11B*. New clinical features and immunophenotypes were identified.

## Materials and Methods

### Patient

A 17-month-old girl was admitted to the Hunan Children's Hospital for intellectual disability and developmental delay. The patient with an individual phenotype was suspected to have a congenital disorder caused by gene mutation. Thus, a genetic study focusing on this case was performed. The study was approved by the Ethics Committee of Hunan Children's Hospital (ID: HCHLL-2019-47).

### Clinical Tests

The patient went through multiple clinical examinations, including physical, neurological, and immunological evaluations. Electroencephalogram (EEG), electromyogram (EMG), and brain magnetic resonance imaging (MRI) were performed for neurological examinations. The immunophenotype of the patient was determined by flow cytometry of peripheral blood mononuclear cells (PBMCs). CD4^+^ or CD8^+^ lymphocytes were identified by antibody detection. Naive CD4^+^/CD8^+^ T cells were defined as CD3^+^CD45RA^+^CD62L^+^ cells in the CD4^+^/CD8^+^ subset, and recent thymic emigrants (RTEs) were defined as CD45RA^+^CD31^+^ cells in the CD4^+^ subset. γδ T cells were defined as T cells with a positive TCR-γδ signal.

### Sequencing Analysis

Peripheral blood samples of the proband and her parents were collected and then sent to Running-Gene Inc. (Beijing, China) for genetic analysis including whole-exome sequencing (WES) and Sanger sequencing. DNA samples were isolated using a DNA Isolation kit (Bioteke, China). Genomic DNA was quantified using the Qubit dsDNA HS Assay kit (Invitrogen, Q32851) and fragmented in a Covaris Acoustic System (Covaris, Massachusetts, USA). A DNA library was established using a KAPA Library Preparation kit (Kapa Biosystems, KR0453). DNA fragments were estimated and quantified before captured by the Agilent SureSelectXT2 Target Enrichment System (Agilent, CA, USA). Captured fragments were PCR enriched before sequencing on an Illumina HiSeq X10 platform (Illumina, San Diego, CA, USA) with 150-bp paired-end reads.

We aligned raw data against the human reference genome (GRCh37/hg19) using the Burrows–Wheeler Alignment tool (http://bio-bwa.sourceforge.net/). Single-nucleotide polymorphisms (SNPs), insertions/deletions (indels), and duplicate reads were identified using the GATK software (Genome Analysis ToolKit) (www.broadinstitute.org/gatk). All called variants were annotated by ANNOVAR (annovar.openbioinformatics.org/en/latest/) based on genetic information in public databases (including the 1000 Genomes Project, ExAC, and gnomAD). All mutations were categorized according to the American College of Medical Genetics and Genomics (ACMG) guidelines (8).

The candidate causal variants identified via WES were further confirmed by Sanger sequencing together with cosegregation analyses. Primers were designed using Primer Premier 5.0 (Premier Biosoft, CA, USA). Target fragments were PCR amplified, purified, and sequenced by an ABI 3730XL DNA Sequencer (Applied Biosystems, CA, USA). Sequencing results were viewed and analyzed by Chromas Lite v2.01 (Technelysium Pty Ltd., Tewantin, QLD, Australia).

## Results

### Clinical Information

**Figure 1:**
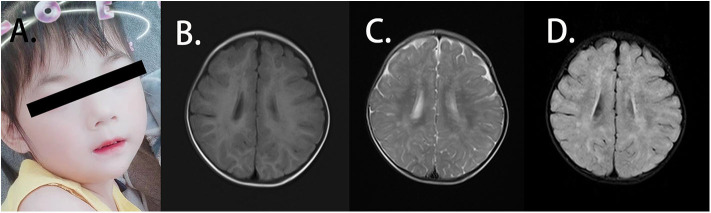
Clinical features of the patient. **(A)** The patient presented with facial dysmorphic features with hypertelorism, long philtrum, and thin upper lip vermilion. **(B)** Axial T1W1 magnetic resonance imaging (MRI) of the patient displayed blotch and piece-like lesions with the prolonged signal. **(C)** Axial T2W1 MRI imaging of the patient showed the abnormal shades distributed in bilateral frontal, temporal, parietal, and occipital lobes and basal ganglia. **(D)** From axial FLAIR imaging, the abnormal lesion was identified mainly in the white matter, and strip-like signals can also be observed in the bilateral basal ganglia.

The patient was the second child of a family with a healthy elder brother. Her parents were not consanguineous and did not have a family history of related diseases. She presented with weak functional movement and hypertonia in all limbs when admitted to our hospital. She could not sit stably or speak. A blood routine test showed normal immunoglobulin levels, and the anti-streptolysin O (ASO) test was negative. The patient was 85 cm in height [−2 standard deviation (SD)], 11 kg in weight (−2 SD to −1 SD) and 47 cm in head circumference (−1 SD) at 29 months. She presented with mild facial dysmorphic features with hypertelorism, long philtrum, and thin upper lip vermilion (Figure 1A). The patient showed slow development in relation to her age. She could not hold head steady at the age of 4 months. At the age of 6 months, she could not sit without support or try to get things that are out of reach. When she was 18 months old, she was unable to walk alone or say several single words. The auditory reflex pathway was established. However, she still could not grasp things by the hands. She was likely to fall back when she sat. She supported herself on a pointy foot when she stood, and she presented with hypertonia in the limb muscles. At the age of 26 months, she could walk with support but easily fell when she walked alone. She had no verbal communication with others, and eye contact was few. Until the age of 45 months, she could walk alone and follow instructions. However, she still had difficulties in speaking and feeding. She never communicated with others actively but could say simple words. No autistic or anxiety features were noted. The patient was previously affected by the EV71 virus before being admitted to our hospital, presenting with mild hand-foot-and-mouth disease with blisters and ulcers in her mouth, according to her medical history. During the infection, the white blood cell level of the patient was always normal. Disease symptoms were resolved without other abnormalities. No additional infections were noted by enterovirus assay. What should be noted is that the guardian of the patient thought the patient had no severe symptoms and could live normally, so the patient was not given any treatments.

### Neurological Examination

EEG and EMG indicated standard findings. Symmetrical abnormal lesions were identified in the bilateral frontal, temporal, parietal, and occipital lobes as well as the basal ganglia by brain MRI (Figures 1B–D). These lesions were mainly distributed along the myelin, suggesting the abnormal myelination of the white matter.

### Immunophenotype

**Figure 2:**
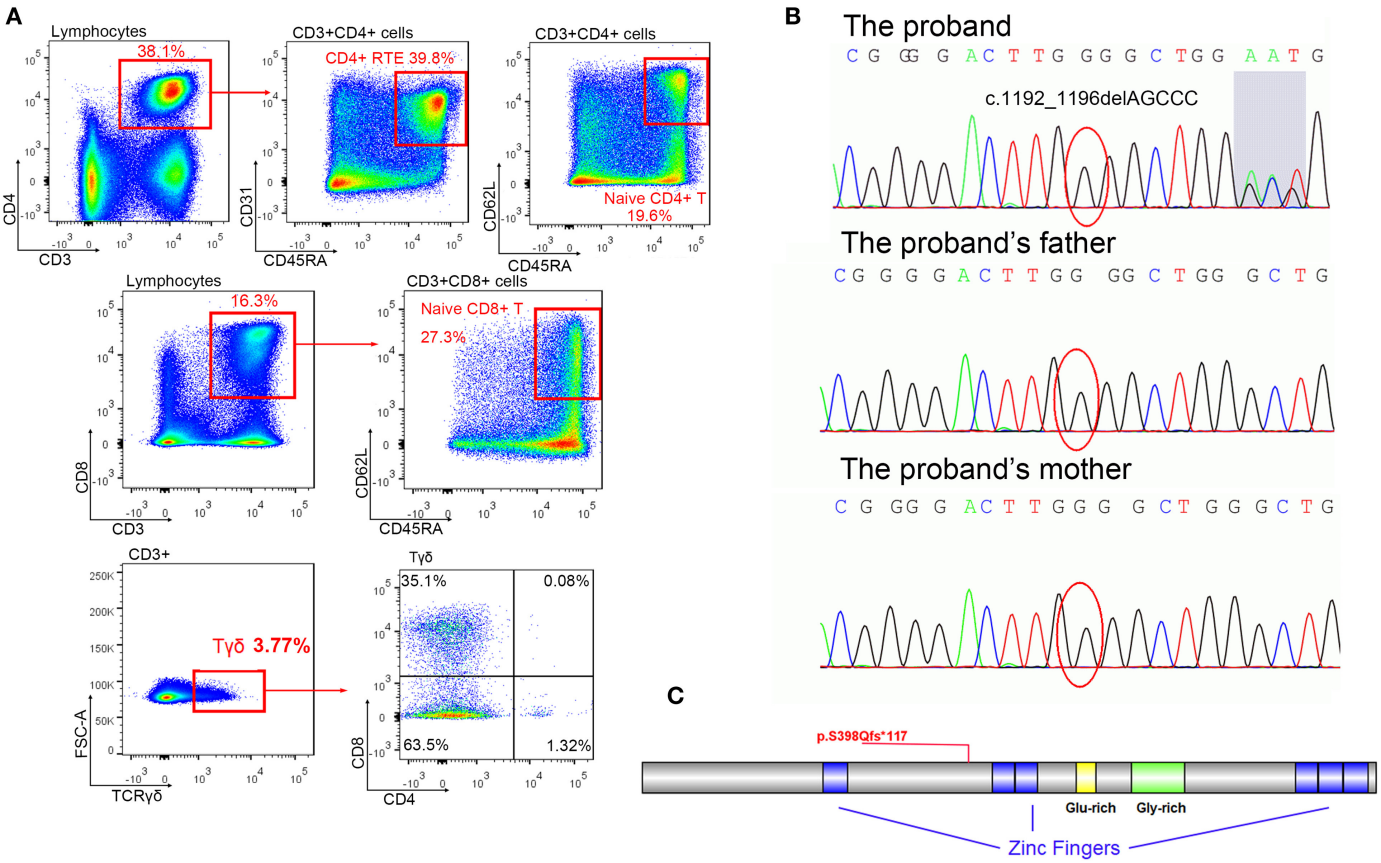
The mutation of *BCL11B*. **(A)** Immune cell analysis of the patient indicated lymphopenia. **(B)** Mutation c.1192_1196delAGCCC was only identified in a patient (shadow shows the chaos of subsequent sequence), but her parents displayed regular sequences. **(C)** The schema of the *BCL11B* gene with the frameshift mutation of this study.

Fresh PBMCs collected from the patient at the age of 18 months were stained and sorted into CD4^+^CD8^+^ conventional T cells. No significant abnormalities were identified. Another set of analyses to sort γδ T cells and naive T cells were performed at the age of 34 months (Figure 2A, Table 1). The percentage of CD8^+^ cells was low normal and not clinically significant. T-cell abnormalities were identified in low numbers of naive CD4^+^ and naive CD8^+^ T cells. High numbers of CD4^−^CD8^−^ double-negative (DN) T cells were observed. Normal numbers of γδ T cells and RTEs were observed in the patient. However, in the γδ T-cell subset, the percentage of CD4^−^CD8^−^ DN cells (63.5%) was lower than expected (normal range >70%), and the percentage of single-positive (SP) cells, including 35.1% CD4^−^CD8^+^ cells (normal range 30%) and 1.32% CD4^+^CD8 CD4^−^ cells (normal range <1%), was slightly higher than normal (9). Considering the immune abnormalities and neurodevelopment delay, this patient was clinically diagnosed as IDDSFTA.

**Table 1:** Immunophenotype of the patient.

**Cells**	**18 months**	**34 months**	**Normal range**
T cells (%)	70.48	61.9	58.7–75^*^
T cells (cells/μl)	1,827	1,383	1,000–3,300^#^
CD3^+^CD4^+^ (%)	42.35	38.1	28.3–46.5^*^
CD3^+^CD4^+^ (cells/μl)	1,098	851	500–1,600^#^
CD3^+^CD8^+^ (%)	20.75	16.3	16–29.5^*^
CD3^+^CD8^+^ (cells/μl)	538	364	320–1250^#^
CD3^+^CD4^−^CD8^−^ (%)	NA	12.2	<7^§^
CD4/CD8 ratio	2.04	2.34	1.06–2.54^*^
Naïve T in CD4^+^ (%)	NA	19.6	50–70^§^
RTE in CD4^+^ (%)	NA	39.8	about 40^§^
Naïve T in CD8^+^ (%)	NA	27.3	50–90^§^
γδT in CD3^+^ (%)	NA	3.77	<6^§^
CD4^−^CD8^−^ in γδT (%)	NA	63.5	>70^§^
CD4^+^CD8^−^ in γδT (%)	NA	1.32	<1^§^
CD4^−^CD8^+^ in γδT (%)	NA	35.1	~30^§^

*Values higher than the normal range were marked in red, and levels lower than the normal were highlighted in blue*.

* *The reference ranges of peripheral blood lymphocyte subpopulations for male Chinese children aged from 12 to 36 months old (n = 116)*.

# *The reference ranges of peripheral blood lymphocyte subpopulations for normal Chinese infants of our hospital aged from 12 to 24 months old*.

§ *The reference ranges suggested by previous publications (7, 9–11)*.

### Sequencing Results

A *de novo* heterozygous mutation c.1192_1196delAGCCC (chr14: 99641977-99641981, NM_138576.2) was identified in *BCL11B* of the patient, resulting in a frameshift mutation p.S398Qfs^*^117 that could produce truncated proteins (Figures 2B,C). The messy tail of the mutant protein was also analyzed, but no functional domain was coincidently established. Both parents and her elder brother carried wild-type *BCL11B*. This mutation has not been recorded in the genome databases of healthy controls (including ExAC and gnomAD) or reported in the Human Gene Mutation Database (HGMD). *In silico* algorithms such as MutationTaster2 (14) also predicted this variation as damaging. According to the ACMG guidelines, this mutation was classified as pathogenic.

## Discussion

*BCL11B* is mapped to chromosomal 14 and encodes an 894-amino acid transcription factor that might control the expression of multiple immune promoters or receptors by binding to the coding frame of genes (7). Six zinc finger structures were identified as the functional domains. This protein was identified to be essential for modulating the expressions of CCR7 and CCR9 receptors and directing the movement of progenitors and mature lymphocytes (12). *BCL11B* might also be associated with the proliferation, migration, and differentiation of neural stem cells, neurons, and granule cells (15). Mutant *BCL11B* was associated with two types of neurodevelopmental disorder and T-cell deficiency (7, 12). However, limited numbers of patients with various clinical phenotypes have been reported, and the pathogenic mechanism and detailed distinctions are still unclear. To consolidate our understanding of *BCL11B*-associated disease, we report a patient with neurodevelopmental delay and naive T-cell deficiency, who presented with a unique phenotype and carried a novel *BCL11B* mutation.

A *de novo* heterozygous mutation c.1192_1196delAGCCC in *BCL11B* was identified in this patient. The truncating protein only retains 398 amino acids of the correct sequence with one zinc figure domain (Figure 2C), indicating that it probably lacks DNA binding capacity and would fail to modify the expression of multiple receptors. The increased numbers of CD4^−^CD8^−^ DN cells observed in this patient were consistent with the immunophenotype of *BCL11B*-knockout mice (3), indicating a loss-of-function mutation in this patient.

There are 15 IMD49 and IDDSFTA patients carrying the heterozygous *BCL11B* mutations that have been reported until now (Table 2). Eight frameshift mutations (p.Cys81Leufs^*^76, p.Thr502Hisfs^*^15, p.Arg518Alafs^*^45, p.Asp534Thrfs^*^29, p.Gly649Alafs^*^67, p.Thr730Thrfs^*^151, p.Gly820Alafs^*^27, and p.Ala891Profs^*^106), two nonsense mutations (p.Try455^*^ and p.Glu499^*^), two missense mutations (c.1323T>G, p.Asp441Lys and c.2421C>G, p.Asn807Lys), and two chromosomal rearrangements [46, XY, t(4;14) (p15;q32.1); 46, XY, t(4;14) (q31.1;q32.2)] are reported to result in diminished *BCL11B* expression, leading to different clinical characteristics of patients. Compared with other reported patients, our patient (c.1192_1196delAGCCC) presented with typical features, including movement disorder, language delay, and immunodeficiency. Some special features, such as dental or skin abnormalities, atopy, eosinophilia, autistic, or anxiety features that have been reported in some patients, were not observed in our patient. However, the MRI of our patient was abnormal. Previously, only three patients displayed abnormal MRI, who had a moderate ectopia of amygdala, hypoplasia of the globus pallidus (7), or callosal agenesis (12). Our patient presented with abnormal myelination pattern of white matter, which has not been reported in IMD49 or IDDSFTA patients. Previous investigations suggested that structural viral protein 1 of EV71 might promote autophagy of myelin cell and induce encephalitis, which resulted in fatal neuronal damage (16). Considering the normal white blood cell level of the patient during EV71 virus infection, we excluded encephalitis and neuronal damage caused by the EV71 virus (17). Thus, our case suggests that the damage of brain white matter might be associated with the absence of functional BCL11B.

**Table 2:** Variants and clinical manifestations of patients harboring *BCL11B* mutations.

**Case**	**Variant**	**Sex**	**Intellectual disability**	**Speech impairment**	**Delay in motor development**	**Autistic features**	**Myopathic facial appearance**	**Thin eyebrows**	**Small palpebral fissures**	**Hypertelorism**	**Prominent nose**	**Long philtrum**	**Thin upper lip vermilion**	**Refractive error**	**Dental anomalies**	**Feeding difficulties**	**Abnormal MRI**	**Immune response**	**Allergy/asthma**
		**6F/9M**	**15/15**	**15/15**	**14/15**	**4/15**	**6/15**	**7/15**	**9/15**	**10/15**	**11/15**	**12/15**	**14/15**	**5/15**	**6/15**	**3/15**	**4/15**	**8/15**	**8/15**
1. Present	c.1192_1196delAGCCC p.S398Qfs*117	Female	+	+	+	–	–	–	–	+	–	+	+	–	–	–	+	Deficiency of naïve T-cells	–
2. Lessel et al. (7)	c.242delG p.C81Lfs*76	Male	+	+	+	–	–	–	–	–	+	–	+	Myopia	+	–	–	Frequent/atypical infections	+
3. Punwani et al. (12)	c.1323T>G p.N441K	Male	+	+	+	–	–	+	+	+	+	+	+	–	+	–	+	Low TREC at birth	+
4. Lessel et al. (7)	c.1365_1367delCAA p.Y455*	Male	+	+	+	–	–	–	–	+	–	+	+	Exotropia	–	–	–	–	+
5. Lessel et al. (7)	c.1495G>T p.E499*	Female	+	+	+	–	+	–	+	–	–	+	–	Myopia	–	+	+	–	+
6. Lessel et al. (7)	c.1502dupG p.T502Hfs*15	Female	+	+	+	–	+	–	–	+	–	+	+	–	–	–	–	–	–
7. Lessel et al. (7)	c.1552delC p.R518Afs*45	Male	+	+	+	–	–	+	–	+	+	+	+	–	+	–	–	Frequent infections^a^	+
8. Lessel et al. (7)	c.1600delG p.D534Tfs*29	Female	+	+	+	+	+	+	+	+	+	+	+	–	–	–	–	–	–
9. Lessel et al. (7)	c.1944_1965delGGCGCGGTCAACGGGCGCGGGG p.G649Afs*67	Male	+	+	+	–	–	+	+	–	+	+	+	–	+	–	–	Frequent infections^a^	–
10. Qiao et al. (13)	c.2190_2200delGGACGCACGAC p.T730Tfs*151	Female	+	+	+	–	–	+	+	+	+	+	+	–	–	–	–	Low TREC at birth	–
11. Lessel et al. (7)	c.2421C>G p.N807K	Male	+	+	+	–	+	–	+	+	+	–	+	–	+	+	–	Low TREC at birth	+
12. Lessel et al. (7)	c.2449_2456dupAGCCACAC p.G820Afs*27	Female	+	+	+	+	+	+	+	+	+	+	+	Hyperopia	+	–	–	–	–
13. Lessel et al. (7)	c.2671delG p.A891Pfs*106	Male	+	+	+	+	+	+	+	+	+	+	+	Hyperopia	–	+	–	Frequent infections^a^	+
14. Lessel et al. (7)	46,XY,t(4;14) (p15;q32.1)	Male	+	+	+	+	–	–	–	–	+	–	+	–	–	–	+	–	+
15. Lessel et al. (7)	46,XY,t(4;14) (q31.1;q32.2)	Male	+	+	–	–	–	–	+	–	+	+	+	–	–	–	–	–	–

Oligodendrocytes, the myelin-forming cells, are thought to differentiate from neural stem cells via oligodendrocyte precursor cells (18, 19). Many cytokines were reported to participate in the differentiation of oligodendrocytes (20), including cyclin-dependent kinase inhibitor p27/Kip1, which increased the differentiation of oligodendrocytes from induced pluripotent stem cells (21). *BCL11B* was reported to control hippocampal neurogenesis by regulating cyclin-dependent kinase inhibitor levels in proliferating progenitors (15). Therefore, *BCL11B* might have a role in the differentiation of oligodendrocytes, whereas mutant *BCL11B* might affect myelination and cause the damage of brain white matter.

When comparing the immunophenotype of our patient with all reported patients, we noticed that the low numbers of naive T cells in our patient was similar to most reported patients. However, our patient still presented with unique immune features that were inconsistent with previously reported patients. A comparison of the immunophenotypes of reported patients indicated that all patients had abnormally high levels of γδ T cells, except that one patient presented with unusually low RTE levels (7, 12). We report the first IDDSFTA patient with normal percentages of γδ T cells and RTE. Our case also showed the lowest CD8^+^ T cell level to date. These results extend the immunophenotype spectrum of IDDSFTA.

Typically, RTE represents a group of young CD4^+^ T cells with undiluted copies of T-cell receptor excision circles (TREC) (22). γδ T cells and RTE values of our patient indicated a normal output of thymuses after the rearrangement of TCR. Low numbers of naive T cells suggested that fewer cells managed to pass central tolerance selection in the thymus. The increased number of CD4/CD8 SP cells in the γδ T subset indicated that the TCR formation pathways were normal. *BCL11B* controls the expression of CCR7/CCR9 in hematopoietic progenitor cells (12), and CCR7 is required for the negative selection of autoreactive thymocytes in the thymic medulla (23). Therefore, *BCL11B* might also have a role in the negative selection procedure: the mutant *BCL11B* might affect negative selection during central tolerance selection and decrease the naive T-cell output.

Considering the pathogenicity of the *BCL11B* mutation in our patient, ILC2 levels were predicted to be reduced, which would increase the risk of respiratory diseases or dermatitis in the future. However, we were not able to perform an ILC assay to detect ILC2 levels in our patient. Further investigations are required to enhance our understanding of the disease and identify factors that contribute to the clinical phenotypes of the disease. More comprehensive care should also be performed on the patient for a better prognosis.

In conclusion, we report an IDDSFTA patient from East Asia with unique phenotypes. We have extended the spectrum of the clinical features and genotypes of IDDSFTA, which will contribute to the diagnosis and understanding of *BCL11B*.

## Data Availability Statement

The original contributions presented in the study are included in the article, fastQ data have been uploaded into the NCBI SRA database (ID: PRJNA659874).

## Ethics Statement

The studies involving human participants were reviewed and approved by Ethics Committee of Hunan Children's Hospital. Written informed consent to participate in this study was provided by the participants' legal guardian/next of kin. Written informed consent was obtained from the individual(s), and minor(s)' legal guardian/next of kin, for the publication of any potentially identifiable images or data included in this article.

## Author Contributions

ZX investigated the subject and conceived the target. SY designed the study, performed the experiment, and drafted the immunology part of the manuscript. QK enrolled and examined the patient and wrote the neurology part of the manuscript. YH applied the genetic analysis and drafted the genetic portion of the manuscript. LW and LL performed the flow cytometry analysis. SL collected clinical information from the patient and her family and recorded data. HL collected information on reported patients and summarized disease characteristics. ZC supervised the sequencing analysis. LY and ZX reversed the final manuscript. All authors contributed to the article and approved the submitted version.

## Conflict of Interest

YH and ZC were employed by the company Running Gene Inc. The remaining authors declare that the research was conducted in the absence of any commercial or financial relationships that could be construed as a potential conflict of interest.

## Abbreviations

IMD49: immunodeficiency 49
IDDSFTA: intellectual developmental disorder with speech delay, dysmorphic facies, and T-cell abnormalities
EEG: electroencephalogram
EMG: electromyogram
MRI: magnetic resonance imaging
PBMCs: peripheral blood mononuclear cells
ASO test: anti-streptolysin O test
DN: double-negative
SP: single positive
DP: double positive
RTEs: recent thymic emigrants
HGMD: Human Gene Mutation Database
ACMG: American College of Medical Genetics and Genomics
TREC: T-cell receptor excision circles
TCR: T-cell receptor.
